# The Mutant Thyroid Hormone Receptor Beta R320P Causes Syndrome of Resistance to Thyroid Hormone

**DOI:** 10.1155/2018/4081769

**Published:** 2018-12-31

**Authors:** Tetsuya Kimura, Yoshitaka Hayashi, Yuka Tsukamoto, Yasuyuki Okamoto

**Affiliations:** ^1^Okamoto Thyroid Clinic, Asahi-ku, Osaka 535-0031, Japan; ^2^Research Institute of Environmental Medicine, Nagoya University, Aichi 464-8601, Japan

## Abstract

A 31-year-old Japanese male patient with a history of atrial fibrillation showed elevated serum levels of free thyroxine and triiodothyronine and a normal level of thyrotropin. The same abnormal hormone pattern was also found in his son. These data indicated that the index patient and the son have thyroid hormone resistance syndrome. Exon sequencing using DNA from these two patients revealed that both patients harbored a heterozygous mutation in the THRB gene: G1244C in exon 9, which results in R320P substitution. Therefore, thyroid hormone resistance syndrome caused by THRB mutation (RTH*β*) was diagnosed. The mutation of the 320^th^ arginine to proline has not been found to date. In conclusion, herein, we have described the first case of RTH*β* that is associated with R320P mutation.

## 1. Introduction

Thyroid hormone is required for metabolism and physiological functions in various organs such as the heart, brain, liver, and bone. Excess of thyroid hormone activity results in increased heart rate, increased nerve irritability, increased energy consumption, and osteoporosis. To exert its functions, thyroid hormone needs to bind to thyroid hormone receptor alpha (TR*α*) or beta (TR*β*). The syndrome of resistance to thyroid hormone (RTH) is a pathologic condition in which patients show decreased sensitivity to thyroid hormone due to gene mutations [1]. In most cases of RTH, mutations occur in TR*β*. TR*β* that is expressed in the pituitary gland determines the set point of the serum free thyroxine (T4) level. Therefore, the decreased sensitivity of TR*β* results in the compensatory increase of serum thyroid hormone level. As a result, in tissues that express TR*β* predominately, decreased thyroid hormone sensitivity is balanced by the increased thyroid hormone. However, the increased thyroid hormone exhibits excessive hormonal action in tissues that express TR*α*, which has normal thyroid hormone sensitivity. Signs and symptoms of RTH include short stature, attention deficit disorder, and resting tachycardia [2]. The incidence of RTH is estimated to be 1 per 40,000–50,000 live births [3]. Most RTH cases show heterozygous* THRB* mutation and autosomal dominant inheritance. In clinical settings today, RTH is often suspected from abnormal results of thyroid function tests. Elevated serum levels of free thyroxine T4 and/or free triiodothyronine (T3) and unsuppressed thyroid-stimulating hormone (TSH) level are the cardinal features of this entity [4].

In this article, we report a familial case of RTH that is caused by the novel* THRB* mutation, R320P.

## 2. Case Presentation

A 31-year-old Japanese male patient visited our clinic to seek an expert opinion from a thyroidologist. His medical history includes atopic dermatitis and atrial fibrillation, for which he had received cardiac catheter ablation when he was 21 and 25 years old. Although his elevated serum levels of thyroid hormones were apparent at the age of 27, the precise cause had not been identified. The patient was 168 cm tall and weighed 64.8 kg (body mass index was 23.0 kg/m^2^; the ideal body weight for his height is 62.1 kg). His blood pressure was 137/79 mmHg and pulse rate was 115/min, which were regular. His laboratory data showed elevated serum levels of free T4 and free T3 and a normal level of TSH. Autoantibodies for thyroglobulin and TSH receptor were negative. Ultrasonography revealed diffuse goiter (28 ml in volume), which shows homogeneous isoechogenicity. In routine blood tests, serum levels of lipid, protein, and electrolytes were within normal ranges (Table 1).

**Figure 1:**
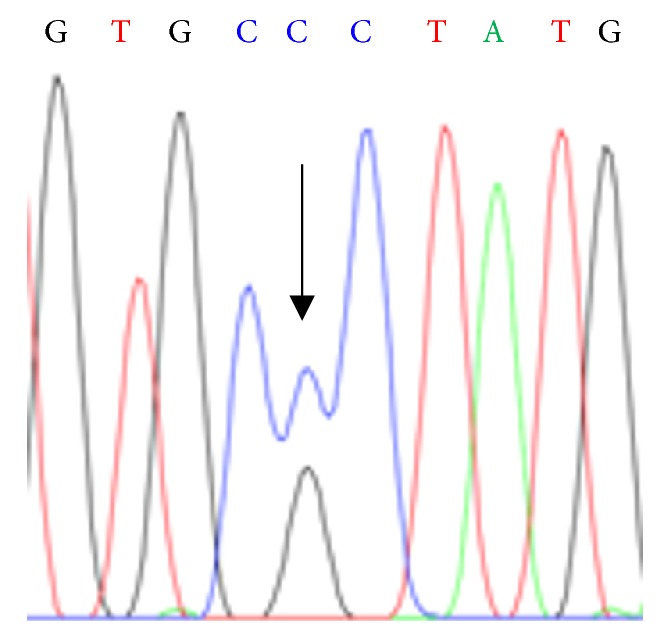
Exon sequencing revealed that a heterozygous point mutation G1244C occurred in the* THRB* gene of the index patient and his son. This mutation leads to a R320P substitution in the TR*β* protein.

Because his 33-month-old son also showed elevated serum levels of free T4 and free T3 and a normal level of TSH (Table 1), we suspected that they had RTH; therefore, we examined sequences of their* THRB* genes. Both the index patient and his son presented with the same heterozygous germline mutation in the* THRB* gene: the 1244^th^ guanine was changed to cytosine (Figure 1). This point mutation results in the substitution of the 320^th^ wild-type amino acid residue arginine to proline. We could not further examine other family members, because the parents of the index patient had died and his brother and sister could not be contacted.

## 3. Materials and Methods

### 3.1. DNA Extraction and Sanger Sequencing

Informed consent was obtained from the patients. Genomic DNA was extracted from peripheral white blood cells. Exons 4 to 10 of the* THRB* gene were sequenced using sense and antisense primers that were previously reported [5].

### 3.2. Alignment Analysis

The wild-type and patients' genome sequences were compared using GENETYX (GENETYX Corp., Shibuya-ku, Japan). The human TR*β* coding sequence that was deposited to NCBI [NM_000461.4] was considered wild-type human* THRB*. According to the guideline for the nomenclature of* THRB* gene mutations [6], we determined the position of the mutant nucleotide and amino acid residue.

## 4. Discussion

Humans have two TR genes,* THRA* and* THRB*, which are located in chromosomes 17 and 3, respectively. Processed by alternative splicing, these two genes are expressed as four functional isoforms, namely, TR*α*1, TR*β*1, TR*β*2, and TR*β*3. TR*α*1 is constitutively expressed at the embryonic stage and, in adults, at the highest level in the brain and the lower levels in the kidneys, skeletal muscles, lungs, heart, and liver. TR*β*1 is predominantly expressed in the brain, liver, kidneys, heart, and thyroid gland, TR*β*2 is mainly expressed in the thyrotroph, retina, and cochlea, and TR*β*3 is predominantly expressed in the kidneys, liver, and lungs [2, 7, 8].

More than 2,000 individuals belonging to about 500 RTH families have been reported since the first* THRB* mutation was identified in 1989 [9]. Most of the mutations were observed in three hot spots in the ligand-binding domain (LBD) and activation function 2 (AF2) domain, namely, the 234^th^–264^th^, 316^th^–347^th^, and 429^th^–454^th^ amino acid residues of TR*β* [8]. These hot spots are distributed in exons 7 to 10 of the* THRB* gene. In this report, we showed the novel mutation R320P in TR*β*. As far as we know, this mutation has not been reported to date. Arginine is a hydrophilic amino acid that has a long side chain; in contrast, proline is a hydrophobic amino acid that has a small side chain containing a 5-carbon ring. The 320^th^ arginine residue is located in the LBD of the TR*β* protein. Other mutations at the 320^th^ arginine, namely, R320L [5], R320H [10], and R320C [10], were previously reported.

Since the molecular elucidation, RTH had almost always been used to describe a condition involving a mutation in the* THRB* gene; however, mutations in other genes have been found recently. In addition to* THRB*, a small fraction of RTH cases are caused by mutations in the monocarboxylate transporter 8 (*MCT8*) [11, 12] and SECIS-binding protein 2 (*SBP2*) genes [13]. Furthermore, resistance to thyroid hormone by heterozygous mutations in* THRA* (currently termed “RTH*α*”) was first reported in 2012 [14, 15]. The symptoms of RTH*α* include bradycardia, neurodevelopmental delay, skeletal dysplasia, dysmorphia, and constipation [14, 15]. Recent discoveries of these new mutations in RTH urged thyroidologists to revise the nomenclature of RTH; according to this revision, RTH*α* and RTH*β* are proposed as the names of RTH caused by mutations in* THRA* and* THRB*, respectively [4].

## 5. Conclusion

In conclusion, we described the R320P mutation in a familial case of RTH*β*. As far as we know, the proline substitution for the wild-type arginine has not been reported to date.

## Figures and Tables

**Table 1:** Laboratory data of the index patient and his son. The son's data are indicated in italic. Data beyond normal ranges are underlined. Normal ranges of thyroid function tests in adults are shown in brackets.

**CBC**				**Thyroid function tests**	
Leukocytes (×10^2^/mm^3^)	112	Albumin (g/dL)	3.8	TSH (*μ*IU/mL) [0.4-4]	1.982
Neutrophils (%)	59.0	AST (IU/L)	15	Free T3 (pg/mL) [2.1-3.9]	4.99
Eosinophils (%)	16.1	ALT (IU/L)	13	Free T4 (ng/dL) [0.85-1.85]	2.76
Basophils (%)	0	*γ*-GTP (IU/L)	18	TRAb (IU/L) [<0.9]	0
Lymphocytes (%)	17.8	LDL-Cho (mg/dL)	97	TgAb (IU/mL) [<5]	0.51
Monocytes (%)	7.1	Triglyceride (mg/dL)	92		
Erythrocytes (×10^4^/mm^3^)	518	HDL-Cho (mg/dL)	41		
Hemoglobin (g/dL)	14.7	BUN (mg/dL)	14.9	**Thyroid function tests (son)**	
Hematocrit (%)	44.1	Creatinine (mg/dL)	0.8	*TSH (μIU/mL)*	*3.013*
Platelets (×10^4^/mm^3^)	37.6	eGFR (ml/min)	95.1	*Free T3 (pg/mL)*	*6.18*
		Na (mEq/L)	137	*Free T4 (ng/dL)*	*2.95*
**Biochemistry**		K (mEq/L)	4.1		
Total protein (g/dL)	7.3	Cl (mEq/L)	100		
